# Contextual Features and Information Bottleneck-Based Multi-Input Network for Breast Cancer Classification from Contrast-Enhanced Spectral Mammography

**DOI:** 10.3390/diagnostics12123133

**Published:** 2022-12-12

**Authors:** Xinmeng Li, Jia Cui, Jingqi Song, Mingyu Jia, Zhenxing Zou, Guocheng Ding, Yuanjie Zheng

**Affiliations:** 1 School of Information Science and Engineering, Shandong Normal University, Jinan 250358, China; 2 Department of Radiology, Yantai Yuhuangding Hospital, Yantai 264001, China

**Keywords:** breast cancer classification, contrast-enhanced spectral mammography, deep learning, contextual features, information bottleneck

## Abstract

In computer-aided diagnosis methods for breast cancer, deep learning has been shown to be an effective method to distinguish whether lesions are present in tissues. However, traditional methods only classify masses as benign or malignant, according to their presence or absence, without considering the contextual features between them and their adjacent tissues. Furthermore, for contrast-enhanced spectral mammography, the existing studies have only performed feature extraction on a single image per breast. In this paper, we propose a multi-input deep learning network for automatic breast cancer classification. Specifically, we simultaneously input four images of each breast with different feature information into the network. Then, we processed the feature maps in both horizontal and vertical directions, preserving the pixel-level contextual information within the neighborhood of the tumor during the pooling operation. Furthermore, we designed a novel loss function according to the information bottleneck theory to optimize our multi-input network and ensure that the common information in the multiple input images could be fully utilized. Our experiments on 488 images (256 benign and 232 malignant images) from 122 patients show that the method’s accuracy, precision, sensitivity, specificity, and f1-score values are 0.8806, 0.8803, 0.8810, 0.8801, and 0.8806, respectively. The qualitative, quantitative, and ablation experiment results show that our method significantly improves the accuracy of breast cancer classification and reduces the false positive rate of diagnosis. It can reduce misdiagnosis rates and unnecessary biopsies, helping doctors determine accurate clinical diagnoses of breast cancer from multiple CESM images.

## 1. Introduction

Breast cancer is a common fatal disease that threatens women’s health [1,2]. Early detection is key to reducing breast cancer mortality [3,4]. However, the diagnostic accuracy in the clinic depends on the physician’s experience [5]. Therefore, using computer-aided diagnosis (CAD) technology to classify breast cancer is of great clinical significance.

The clinical diagnosis of breast cancer is currently based on ultrasound (US), magnetic resonance imaging (MRI), and mammography (MG). Compared with the other methods, US does not show small lesions clearly, and performing retrospective analysis is difficult. MRI is highly accurate but relatively expensive, and its clinical utility is limited in some underdeveloped areas. As an emerging imaging technology, CESM has comparable performance to and is less expensive than MRI in diagnosing breast cancer.

**Figure 1:**
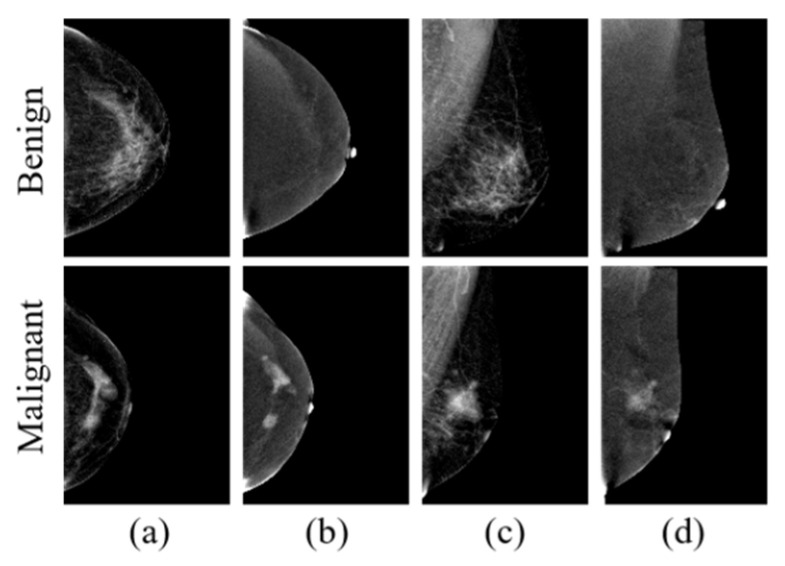
Examples of contrast-enhanced spectral mammography images: (**a**) craniocaudal low-energy image (CC-LE); (**b**) craniocaudal dual-energy subtracted image (CC-DES); (**c**) mediolateral oblique low-energy image (MLO-LE); (**d**) mediolateral oblique dual-energy subtracted image (MLO-DES).

Contrast-enhanced spectral mammography (CESM) is a new technology based on traditional mammography [6,7]. Each breast is irradiated with the standard craniocaudal (CC) view and the mediolateral oblique (MLO) view. Low-energy images (LE), similar to mammography, and dual-energy subtracted images (DES) showing abnormal vascular proliferation in tumor tissue can be generated [8,9]. Figure 1 shows examples of CESM images. CESM has achieved superior diagnostic performance to traditional mammography [10,11,12,13,14].

We introduce existing breast image classification methods, including machine learning and deep learning methods, in Section 2. However, most pay no attention to the neighborhood of the tumor in the breast image. In fact, in natural image processing, contextual features can help identify complex scenes and improve classification accuracy. Similarly, they are also of great importance to medical image processing. Several researchers have conducted experiments and discussed their principles and implications [15,16,17,18,19]. In addition, after each CESM examination, four images with different meanings corresponding to each breast can be obtained. The LE and DES images can display different breast tissue characteristics, and the CC and MLO images can provide the lesion location from different perspectives. It is important to consider features from multiple images and screen out useful information for breast cancer classification.

In order to fully exploit the special feature information of CESM images, we propose a new deep learning classification method. We simultaneously input four CESM images (CC-LE, CC-DES, MLO-LE, and MLO-DES) into the network. Then, we processed the feature maps in horizontal and vertical directions, preserving the pixel-level contextual information within the tumor neighborhood. Then, according to the information bottleneck theory, the common information between them is maximized to obtain more accurate classification results. The main contributions of this work can be summarized as follows.

1. We designed a feature extraction module for accurately discriminating between benign and malignant masses with pixel-level location information in horizontal and vertical directions. This module can capture the contextual features between the lesion area and its adjacent breast tissue, making the network pay more attention to the edge features of the lesion area.

2. We proposed a multi-input CESM image classification network to classify breast cancer. Multiple CESM images are simultaneously input into the network to use complementary features under different views and irradiation energies.

3. We designed a feature selection module, according to the information bottleneck theory, by maximizing the common information between the multiple input images and discarding the irrelevant information from the classification task. We also designed a novel loss function to optimize our multi-input network.

In Section 2 of this paper, we review recent work on breast cancer image classification, especially CESM image classification. In Section 3, we introduce the methodology of the proposed method and details on each module. We also describe the experimental data and parameter settings. We present the results of the qualitative, quantitative, and ablation experiments in Section 4. In Section 5, we discuss the experimental results, implications, and limitations in depth. Finally, in Section 6, we summarize our work.

## 2. Related Work

In recent years, CAD technology has played a significant role in diagnosing breast cancer, which helps improve the accuracy of diagnosis by radiologists [20]. Ragab et al introduced a CAD system based on two feature selection methods for distinguishing normal and abnormal lesions in mammograms [21]. Witowski et al. compared the diagnostic accuracy of a deep learning system with radiologists, reducing the biopsy rate of benign masses [22]. Xu et al. developed a radiomics approach to assist diagnosis on multimodal ultrasound images [23]. Liew et al. used deep learning techniques to classify breast cancer histology images into eight categories [24]. Michael et al. proposed a method for breast cancer detection, based on a decision tree algorithm, and a LightGBM classifier, based on ultrasound images [25].

CESM is an effective breast cancer screening method based on conventional mammography. Recently, there have been several studies on breast cancer classification from CESM images. The methods, datasets, and classification performance of these studies are summarized in Table 1. Marino et al. performed a radiomics analysis based on CESM images [26]. They regarded histopathology as a reference standard and used machine learning methods to describe the morphological features of the breast. Losurdo et al. trained several SVM classifiers to compare the classification performance of different texture feature sets with the overall set [27]. This system extracts regions of interest (ROI) automatically to help radiologists diagnose breast cancer. Danala et al. developed a CAD scheme for classifying breast masses based on CESM images [28]. They constructed MLP classifiers to accurately segment lesions and classify breast cancer. Their method significantly improved the classification performance of CESM images. For breast images, whether masses have irregular shapes or fuzzy edges is one of the significant criteria for judging breast cancer [29,30]. However, in CESM images, people only focus on whether there are masses and ignore many pixel-level features on the edge of the lesion area, and thus may increase the possibility of misdiagnosing benign masses as malignant tumors.

Several research groups then investigated the feasibility of using the differences between CESM and traditional mammography images for breast cancer classification. For example, Gao et al. developed an SD-CNN network to classify CESM images [31]. They trained shallow and deep networks from 49 cases. Their method proved the role of DES images in breast cancer classification. Fanizzi et al. proposed an automatic method to improve the performance of breast cancer diagnosis through CESM images [32]. They used different methods to extract the information of key areas from LE and DES images and trained random forest classifiers. Their research suggested that the proposed method could assist radiologists in detecting breast cancer. Perek et al. improved two networks to classify breast masses in CESM images [33]. They combined textual features with the images’ characteristics and compared the feature fusion and decision fusion methods. Their proposed multimodal network improved the classification performance and reduced the rate of benign biopsy. Dominique et al. used the CheXNet-based deep learning model and tested it on the CESM dataset [34]. They used majority voting rules to calculate the results of images with different characteristics. Their work showed the importance of deep learning technology in CESM. Zhang et al. proposed a breast cancer classification method with multimodal information using RefineNet as the backbone network. Their method pays attention to both CESM images and clinical features, achieving good performance [35]. However, they do not reasonably exploit the CESM images’ unique features. In clinical diagnoses, CC and MLO images show doctors different lesion locations and shape features. These methods ignore information from different illumination views (CC and MLO views).

Our proposed method considers the contextual features between the mass and its adjacent tissues. In addition, we simultaneously use different information from four CESM images to classify breast cancer and obtain common information across multiple images.

## 3. Methods and Materials

### 3.1. The Proposed CESM Classification Method

**Figure 2:**
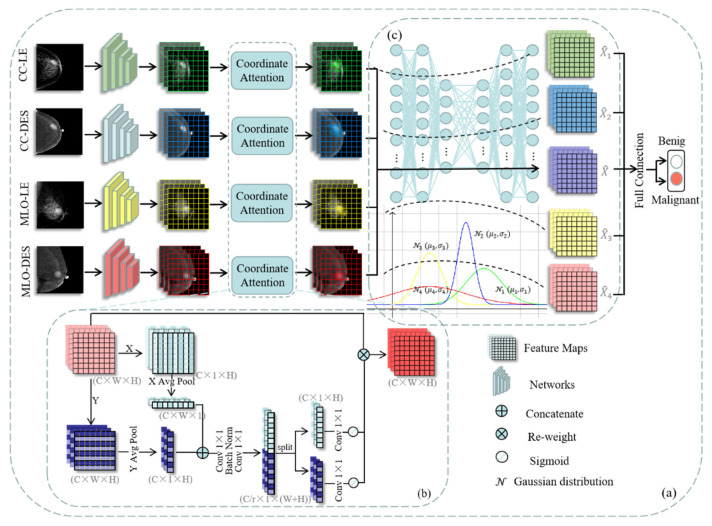
The overview of contextual features and information bottleneck-based multi-input network for breast cancer classification. (**a**) shows the overall flowchart. (**b**,**c**) show the details of the feature extraction module and feature selection module, respectively.

Our method uses ResNet-50 as the backbone and includes a feature extraction module and a feature selection module. Firstly, our network simultaneously receives four images (CC-LE, CC-DES, MLO-LE, and MLO-DES) generated by the CESM detection of each breast. ResNet-50 extracts feature maps corresponding to each input image. Then, the feature maps are input into the feature extraction module. Inspired by the coordinated attention mechanism [36], this module implements pooling operations in the horizontal direction with a pooling kernel of size 1 × 7 and vertically with a pooling kernel of size 7 × 1. The resulting matrices are reweighted onto the original feature maps as the output of the feature extraction module. They are then input into the information bottleneck module, comprising a decoder and an encoder. The decoder and encoder consist of three fully connected layers, extracting the common information from the multiple input images and optimizing the parameters. The features corresponding to the four input images are concatenated and input to a fully connected layer to output the final breast cancer classification result. The flowchart is illustrated in Figure 2a. The feature extraction module in Figure 2b and the feature selection module in Figure 2c are discussed in detail below.

#### 3.1.1. Feature Extraction Module

Global pooling is often used in conventional attention mechanisms to encode spatial information globally, which increases the difficulty of preserving the correlation between the pixels [37]. To overcome the above limitations, we introduce coordinated attention for capturing the pixel-level contextual information between the lesion area and its adjacent breast tissue in a single CESM image input into our classification network.

Given the feature map T, we use the pooling kernel (H, 1) to encode each channel along the horizontal coordinate. Thus, the process can be formulated as

(1) $$T_{i}^{X}\left(i\right)=\frac{1}{H}\underset{0\leq j\leq H}{\sum }t\left(i,j\right),$$

where t refers to the feature map input into the feature extraction module; (i, j) refers to the coordinates of each point in T; and $T^{X}$ is the output of the pooling operation in the horizontal direction. Similarly, the operation of encoding, along with the vertical coordinates with the pooling kernel (1, W), can be written as

(2) $$T_{j}^{Y}\left(j\right)=\frac{1}{W}\underset{0\leq i\leq W}{\sum }t\left(i,j\right).$$

Then, we concatenate the feature maps produced by Equations (1) and (2) and input them together into a 1 × 1 convolutional transformation function $f_{1}$, yielding

(3) $$T_{conv}=ReLU\left(f_{1}\left[T^{X}⊕T^{Y}\right]\right),$$

where [ ⊕ ] denotes the concatenation operation between the two feature maps; ReLU is the non-linear activation function; and $T_{\mathrm{conv}}$ is the output of the 1 × 1 convolutional operation. Here, $T_{\mathrm{conv}}\in {\mathbb{R}}^{\left(H+W\right)\times C/r}$, and r is the reduction ratio for reducing the channel number of $T_{\mathrm{conv}}$ and the model complexity. We then separate $T_{\mathrm{conv}}$ into $T_{\mathrm{conv}}^{X}\in {\mathbb{R}}^{\left(H+W\right)\times C/r}$ and $T_{\mathrm{conv}}^{Y}\in {\mathbb{R}}^{\left(H+W\right)\times C/r}$ in horizontal and vertical directions. $T_{\mathrm{conv}}^{X}$ and $T_{\mathrm{conv}}^{Y}$ are the input into the other two 1 × 1 convolution layers. Finally, the output of the feature extraction module comprises the superposition of the input feature map and the weights obtained in two directions, yielding

(4) $$X\left(i,j\right)=T\left(i,j\right)\times \sigma \left(f_{2}\left(T_{conv}^{X}\right)\right)\times \sigma \left(f_{3}\left(T_{conv}^{Y}\right)\right),$$

where $f_{2}$ and $f_{3}$ denote two 1 × 1 convolutional functions; σ is the sigmoid function; and X(i, j) is the coordinates of each point in the output.

#### 3.1.2. Feature Selection Module

As we have already extracted feature information from multi-input CESM images through the feature extraction module, an effective feature selection method is necessary before the final classification layer to filter out irrelevant features. Mutual information between the layers and the input and output variables can quantify deep neural networks, indicating the relevancy between the information bottleneck and deep learning [38]. The information bottleneck was originally proposed to filter useless information by maximizing the mutual information between objects [39]. With the development of deep learning technology, Tishby et al. discussed the feasibility of combining information bottleneck theory with deep learning tasks [40,41,42]. Therefore, we introduced the information bottleneck theory into our classification method and extended it to multi-input networks.

**Figure 3:**
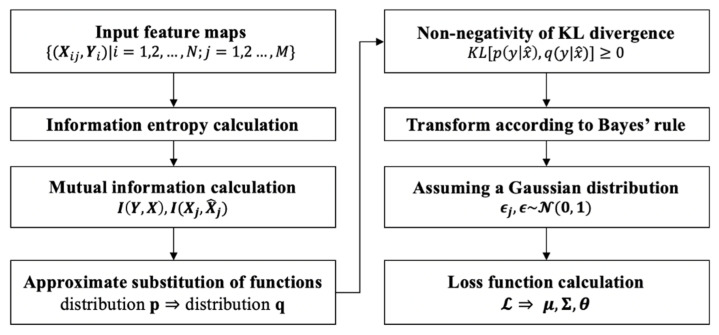
Flow chart of the feature extraction module based on the information bottleneck theory.

The information bottleneck module consists of a decoder and an encoder. The decoder contains three fully connected layers with node numbers 1024, 1024, and 512. Similarly, the encoder contains three fully connected layers with node numbers 512, 1024, and 1024. Each fully connected layer is followed by a ReLU activation layer. We proposed a loss function to train our network, based on the information bottleneck theory. The flowchart of the parameter optimization process is shown in Figure 3.

We input the feature maps’ output by the feature extraction module into the information bottleneck module. In a set containing the feature maps X and ground truth labels Y, $\left\{\left(X_{\mathrm{nm}},Y_{n}\right)|\;n=1,\;2,\;\ldots ,N;m=\;1,\;2,\ldots ,M\right\}$, N and M denote the number of cases and CESM images input into the network simultaneously. According to the information bottleneck theory, the optimization process of the deep learning classification network can be expressed as maximizing the mutual information between the labels and predicted values. In fact, feature maps always contain some information irrelevant to the classification task. Therefore, this process can be formulated as

(5) $$\underset{\hat{X}}{\max}I\left(Y,\hat{X}\right)-\alpha I\left(X,\hat{X}\right),$$

where $\hat{X}$ refers to the relevant part of X with respect to Y, and α is a parameter to trade off the mutual information [43]. I( ; ) refers to the mutual information between the two variables, and it is formulated as

(6) $$I\left(U;V\right)=\int^{\;}p\left(u,v\right)\log\left(\frac{p\left(u|v\right)}{p\left(u\right)}\right)dudv,$$

where p( ) denotes the marginal probability density function, and p( , ) denotes the joint probability density function. Then, we extend it to our multi-input network and learn a joint representation, $\hat{X}$, to optimize our model:

(7) $$\underset{\hat{X},{\hat{X}}_{1},{\hat{X}}_{2},\ldots ,{\hat{X}}_{m}}{\max}I\left(Y,\hat{X}\right)-\overset{m}{\underset{j=1}{\sum }}\lambda_{j}I\left(X_{j};{\hat{X}}_{j}\right),$$

where λ is another form of α in Equation (5), and $f_{\varepsilon }$ is the classification network f with the parameter ε. The first term is to maximize the mutual information between the joint representation $\hat{X}$ and the real label Y. The following items minimize the mutual information between the latent representation of each input image and itself.

Since mutual information is difficult to calculate, we use some known distribution functions to approximate the lower bound of $I\left(Y,\hat{X}\right)$ and obtain the approximate solution. The distribution, p, is complex, whereas the distribution, q, can be learned from the network. Therefore, we use q to approximate p. According to the KL–divergence, we have

(8) $$KL[p(y|\hat{x}),q(y|\hat{x})]\geq 0\;\Rightarrow \int^{\;}dyd\hat{x}\;p\left(y,\hat{x}\right)\;\log\left(p\left(y|\hat{x}\right)\right)\geq \int^{\;}dyd\hat{x}\;p\left(y,\hat{x}\right)\;\log\left(q\left(y|\hat{x}\right)\right).$$

Using Equation (6), we have

(9) $$\begin{array}{c} I\left(Y,\hat{X}\right)\geq \int^{\;}dyd\hat{x}\;p\left(y,\hat{x}\right)\;\log\frac{q\left(y|\hat{x}\right)}{p\left(y\right)}\\ =\int^{\;}dyd\hat{x}\;p\left(y,\hat{x}\right)\;\log q\left(y|\hat{x}\right)-\int^{\;}dy\;p\left(y\right)\;\log p\left(y\right). \end{array}$$

Since the last item of Equation (9) is a definite value that depends on the label y, it has no effect on the parameter optimization. Therefore, we directly drop it and have

(10) $$I\left(Y,\hat{X}\right)\geq \int^{\;}dyd\hat{x}\;p\left(y,\hat{x}\right)\;\log q\left(y|\hat{x}\right)\;=\int^{\;}dyd\hat{x}dx_{1}dx_{2}dx_{3}dx_{4}d{\hat{x}}_{1}d{\hat{x}}_{2}d{\hat{x}}_{3}d{\hat{x}}_{4}\;p\left(x_{1},x_{2},x_{3},x_{4},{\hat{x}}_{1},{\hat{x}}_{2},{\hat{x}}_{3},{\hat{x}}_{4},y,\hat{x}\right)\;\log q\left(y|\hat{x}\right).$$

Using Bayes’ rule, the joint probability density function in Equation (10) can be formulated as

(11) $$\begin{array}{c} I\left(Y,\hat{X}\right)\\ \geq \int^{\;}dx_{1}dx_{2}dx_{3}dx_{4}dy\;p\left(x_{1},x_{2},x_{3},x_{4},y\right)\int^{\;}dyd{\hat{x}}_{1}d{\hat{x}}_{2}d{\hat{x}}_{3}d{\hat{x}}_{4}p\left(\hat{x}|{\hat{x}}_{1},{\hat{x}}_{2},{\hat{x}}_{3},{\hat{x}}_{4}\right)\overset{4}{\underset{j=1}{\prod }}p\left({\hat{x}}_{j}|x_{j}\right)\;logq\left(y|\hat{x}\right). \end{array}$$

We assume that $p\left(\hat{x}|{\hat{x}}_{1},{\hat{x}}_{2},{\hat{x}}_{3},{\hat{x}}_{4}\right)$ and $p\left({\hat{x}}_{j}|x_{j}\right)$ are Gaussian distributions, so we have

(12) $${\hat{x}}_{j}=\mu \left(x_{j};\phi_{j}\right)+\Sigma \left(x_{j};\phi_{j}\right)\epsilon_{j},\;\hat{x}=\mu \left({\hat{x}}_{1},{\hat{x}}_{2},{\hat{x}}_{3},{\hat{x}}_{4};\theta \right)+\Sigma \left({\hat{x}}_{1},{\hat{x}}_{2},{\hat{x}}_{3},{\hat{x}}_{4};\theta \right)\epsilon ,$$

where $\epsilon_{j},\epsilon \sim N\left(0,I\right);$ μ denotes the mean; Σ denotes the variance; and θ is a parameter of the network. They are all learned from our network. Similarly, using distribution, r, to approximate p transforms the last terms of Equation (7), according to the KL-divergence. Therefore, the loss function of the information bottleneck module is

(13) $$max\frac{1}{N}\overset{N}{\underset{\;}{\sum }}\left\{E_{\epsilon }E_{\epsilon_{1}}E_{\epsilon_{2}}E_{\epsilon_{3}}E_{\epsilon_{4}}\log q\left(y|\hat{x}\right)-\overset{m}{\underset{j}{\sum }}\lambda_{j}E_{\epsilon_{j}}\log\frac{p\left({\hat{x}}_{j}|x_{j}\right)}{r_{j}\left({\hat{x}}_{j}\right)}\right\},$$

where N denotes the number of cases; $E_{\epsilon }$ and $E_{\epsilon_{i}}$ denote the expected value. The total loss function is the sum of the traditional classification loss and the information bottleneck loss, which can be formulated as

(14) $$ℒ=\frac{1}{N}\overset{N}{\underset{i=1}{\sum }}H\left({\hat{y}}_{i},y_{i}\right)+max\frac{1}{N}\overset{N}{\underset{\;}{\sum }}\left\{E_{\epsilon }E_{\epsilon_{1}}E_{\epsilon_{2}}E_{\epsilon_{3}}E_{\epsilon_{4}}\log q\left(y|\hat{x}\right)-\overset{m}{\underset{j}{\sum }}\lambda_{j}E_{\epsilon_{j}}\log\frac{p\left({\hat{x}}_{j}|x_{j}\right)}{r_{j}\left({\hat{x}}_{j}\right)}\right\}.$$

### 3.2. Materials

#### 3.2.1. Data and Preprocessing

We collected CESM images from the Yantai Yuhuangding Hospital using the all-digital imaging equipment. For suspicious breast cancer patients aged 21–69, the imaging was performed 2 min after intravenous injection of an iodinated contrast agent (300 mg of iodine/mL, 1.5 mL/kg of body weight, flow rate of 3 mL/s), which is administered to the patient using a low-energy (26–32 kVp) and high-energy (45–49 kVp) X-ray spectrum. Low-energy images and dual-energy subtraction images for each breast were obtained through a specific image reconstruction algorithm at the craniocaudal and mediolateral oblique, with a total of four mammography images. Based on the imaging examination, the clear diagnosis made by doctors through a biopsy is regarded as the standard of our classification task. According to the standard, we divided the image data into two categories, with 64 benign cases and 58 malignant cases. Then, we divide the dataset into the training, verification, and test sets according to the proportion of 80%, 10%, and 10%, respectively. The resolution of all the images is adjusted to 1350 × 2300 px. When working with deep learning, it is crucial to use a large enough dataset to train the model. Especially for medical image processing, data enhancement to generate new data sets is also beneficial to protect the patients’ privacy. We conduct a series of operations on the images in the CESM dataset, such as pan, rotate, flip, and zoom. At the same time, we use a Gaussian Blur to simulate the real noise information.

#### 3.2.2. Details of Training

We evaluate our method based on the data-enhanced CESM dataset. All the images are resized to 227 × 227 px in our experiments. The method proposed in this paper is based on PyTorch implementation, and it is trained on the NVIDIA Tesla A30 GPU. The number of training iterations is 150. We optimize the weights by the ADAM algorithm, with β1 = 0.900. The effects of several super parameters on the method are tested experimentally. When the batch size, learning rate, and β2 are set to 16, 0.001, and 0.999, respectively, the method obtains the best results.

## 4. Results

### 4.1. Qualitative Comparison

**Figure 4:**
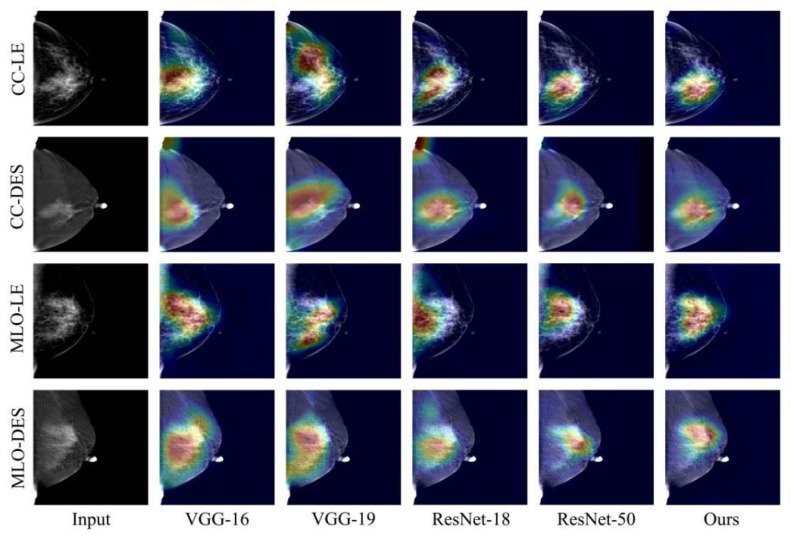
Visual interpretations generated by different methods using Grad-CAM. The first column shows the four input CESM images. The last column shows the visual interpretation generated by our method.

We compare our method to the traditional methods of VGG-16, VGG-19, ResNet-18, and ResNet-50. We use Grad-CAM [44] to visually interpret our method and the others. Figure 4 shows the qualitative experimental results of four CESM images input into the method in the test set. VGG-16 and VGG-19 focus on lesion locations but also on some regions that are not relevant to the breast cancer classification task. ResNet-18 and Resnet-50 produce competitive results, but the details are still not as accurate as our method. Due to the IB feature screening module we designed, our method focuses less on irrelevant features. Our method produces excellent visuals of the location and size of the mass, especially its shape and edge. This effect is mainly due to our well-designed feature extraction module.

### 4.2. Quantitative Comparison

**Figure 5:**
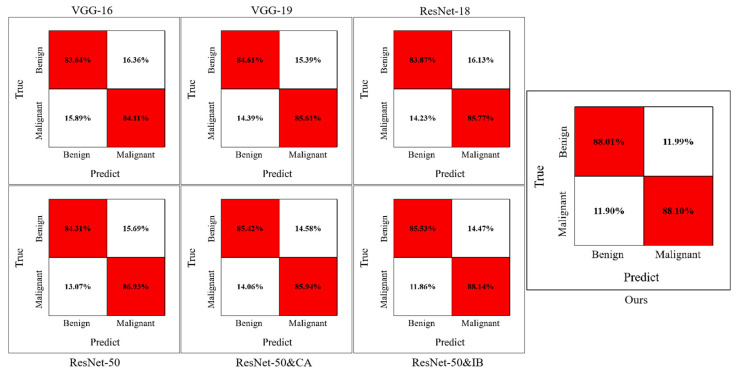
Confusion matrices for the methods in our experiment.

**Figure 6:**
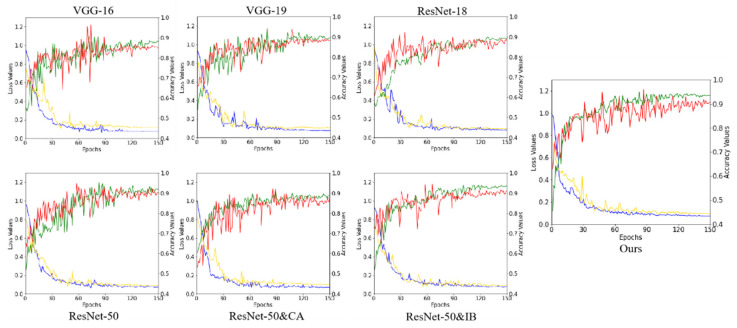
The loss and accuracy of the methods in the experiment. The green line represents the training accuracy. The red line represents the validation accuracy. The blue line represents the training loss. The yellow line represents the validation loss.

**Figure 7:**
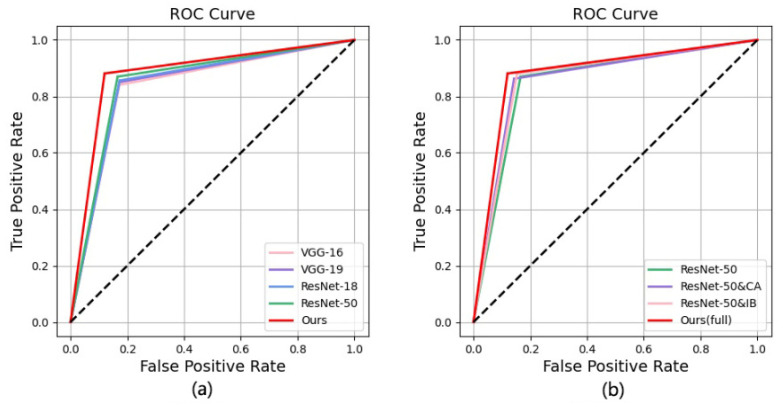
Comparison of ROC curves of different methods. The solid red line represents our method. (**a**,**b**) show the results of our methods in qualitative and quantitative experiments, respectively.

We test the performance of the methods on the CESM testing set and evaluate them by accuracy, precision, sensitivity, specificity, and F1-score. As listed in Table 2, our method achieves higher accuracy and more balanced performance than the other methods. Based on the experimental data, we draw confusion matrices for all the methods. As shown in Figure 5, our method outperforms the traditional methods. In particular, our network misdiagnosed fewer benign cases as malignant. In addition, the receiver operating characteristic (ROC) curve plots further demonstrate the diagnostic power of the binary classifier. Figure 6 shows the loss and accuracy of the methods in the experiments. As shown in Figure 7a, the experimental results are shown through the ROC curve. Our method achieves the highest area under the curve (AUC), a higher true positive rate (TPR), and a lower false positive rate (FPR).

### 4.3. Ablation Studies

**Figure 8:**
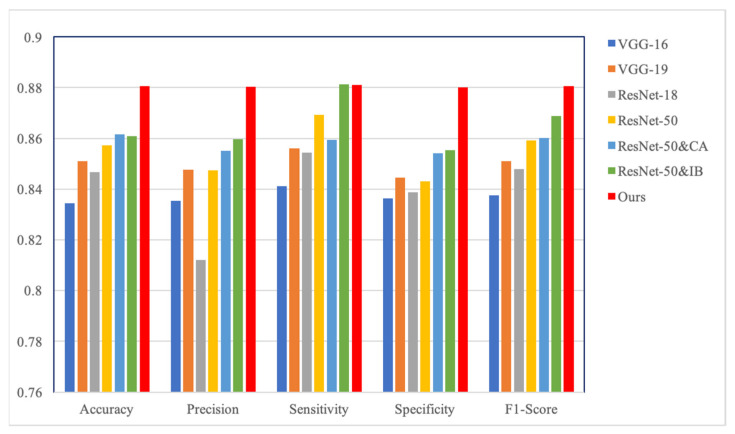
Comparison of classification performance between comparative experiments and ablation studies.

Our method consists of a feature extraction module using the coordinated attention (CA) principle and a feature selection module using the information bottleneck (IB) theory. Therefore, we verified their contribution to the results through experiments: (1) taking ResNet-50 as the backbone, without CA and IB, which is our baseline; (2) introducing only CA into the baseline; (3) introducing only IB into the baseline; and (4) the baseline with CA and IB, which is our method. Table 3 shows the results of CA and IB ablation studies. ResNet-50 and CA has a more balanced performance than the baseline, but its overall performance is lower than our method. Similar to our method, ResNet-50 and IB achieves the best sensitivity, but its performance is not as high in other aspects. As shown in Table 3, our method has more competitive performance when CA and IB are added to the baseline. In Figure 7b, our method achieves a higher area under the curve than the other methods. Figure 5 shows the confusion matrices of the ablation experiments, and Figure 8 shows the performance improvement. The ablation experiment results show that both CA and IB are necessary for improving the performance of the CESM image classification.

## 5. Discussion

In this study, we propose a deep learning method for classifying contrast-enhanced spectral images based on contextual features and the information bottleneck principle. We curate the raw data obtained from the hospital and perform data augmentation. Since each CESM examination produces four images with complementary features corresponding to each breast, we input them into our network simultaneously. In the feature extraction step, we introduce a coordinated attention mechanism that enables our network to capture pixel-level contextual information between the lesions and adjacent breast tissue. Then, we use the information bottleneck theory to perform feature screening on the four feature maps and generalize it into multi-input networks. This process provides our network with a more reasonable loss function for further optimization. Finally, we fuse the feature information from multiple input images to train the network and obtain the final classification of benign or malignant breast cancer.

Previous studies mainly focused on developing classification networks for a single CESM image. Recently, researchers considered the difference between LE and DES images and used both to classify breast cancer. Perek et al. combined text and image features to classify breast masses in CESM images [33]. Dominique et al. considered different LE and DES image features and used a majority voting rule to calculate the results [34]. A recent approach is the two-view convolutional neural network proposed by Sun et al. [45]. They used both CC and MLO views for breast cancer classification but did not consider DES images. In order to fill the previous gap, we use four images (CC-LE, CC-DES, MLO-LE, and MLO-DES) as input and extract the common information between them for the breast cancer classification. Our network considers more comprehensive feature information than the previous methods.

We assess the performance of our proposed method through qualitative and quantitative evaluation in our experiments. Figure 4 shows the interest regions of different methods for a set of input images. VGG-16 and VGG-19 focus on some highlighted areas in CESM images that are irrelevant to breast cancer diagnosis. ResNet-18 and ResNet-50 focus on the tumor location more accurately but also consider irrelevant regions. Therefore, having the network focus less on irrelevant regions is necessary. In addition, Table 2 shows that the commonly used methods achieve higher sensitivity in the breast cancer classification task. It can also be observed from Figure 7 that these methods achieve higher false positive rates. Therefore, we require a method that reduces the misdiagnosis rate of the CESM images. Our proposed feature extraction module enables the network to preserve the pixel-level contextual information of the lesions. This process allows our network to focus on the lesion’s edges and the influence of adjacent tissues, reducing the probability of misdiagnosing benign masses as malignant.

However, most existing studies on CESM image classification conduct experiments on private datasets. Due to medical technology and privacy constraints, there are not many cases in these datasets, and the variety of lesions is not diverse. These deficiencies should be addressed in future works based on this study.

## 6. Conclusions

In this paper, we propose a multi-input classification network based on contextual features and the information bottleneck to correct the high misdiagnosis rate of breast cancer caused by traditional classification methods, which extracts the irrelevant features. We use ResNet-50 as the network backbone to extract features from four CESM images corresponding to the same breast. Our feature extraction module accurately localizes the mass in horizontal and vertical directions and preserves the pixel-level contextual information between the mass and its neighborhood. This process helps the network focus on the impact of the breast tissue surrounding the lesion. We also propose a feature selection module, based on the information bottleneck theory, to filter out the features irrelevant to the classification task in multiple feature maps and preserve their common information. We evaluated the performance of our network using multiple evaluation metrics on a dataset of 488 images from 122 patients. The qualitative, quantitative, and ablation experiment results show that our method significantly improves the accuracy of breast cancer classification and reduces the false positive rate of diagnosis. We plan to collect more CESM images to expand the dataset in future work. This step is beneficial to improving the robustness of our method. Furthermore, we did not consider other image types, such as ultrasound and magnetic resonance imaging data. Our future work will combine different data types from the same breast for breast cancer classification.

## Figures and Tables

**Table 1:** Comparison between methods, datasets, and classification performance of CESM images in previous work.

Method	Dataset			Accuracy	AUC
	Type	Source	Number		
Multilayer Perceptron Classifier (Danala et al., 2018) [28]	LE & DES	Clinical Database of Mayo Clinic Arizona	111	-	0.848
SD-CNN (Gao et al., 2018) [31]	LE & DES	Mayo Clinic Arizona & INbreast	49 & 89	0.900	0.920
Support Vector Machine (Losurdo et al., 2019) [27]	CC-DES & MLO-DES	Istituto Tumori “Giovanni Paolo II”	55	0.800	-
Random Forest Classifier (Fanizzi et al., 2019) [32]	CC-DES & MLO-DES	Istituto Tumori “Giovanni Paolo II”	58	0.825	0.850
Fine-tuning Pretrained AlexNet (Perek et al., 2019) [33]	CC-DES & MLO-DES & text	-	129	0.880	0.897
Radiomics Analysis (Marino et al., 2020) [26]	DES	Tertiary Referral Academic Center	100	-	-
Fine-tuning CheXNet (Dominique et al., 2022) [34]	LE & DES	Henri Becquerel Cancer Center	447	0.874	0.910
RefineNet with XGBoost Classifier (Zhang et al., 2022) [35]	CC-LE & CC-DES	Yantai Yuhuangding Hospital and Fudan University Cancer Center	1355	0.802	0.867

**Table 2:** Classification results of CESM images using different deep learning methods. The bold values represent the best value.

Method	Accuracy	Precision	Sensitivity	Specificity	F1-Score
VGG-16	0.8348	0.8354	0.8411	0.8364	0.8376
VGG-19	0.8511	0.8476	0.8561	0.8461	0.8510
ResNet-18	0.8467	0.8412	0.8547	0.8387	0.8479
ResNet-50	0.8572	0.8474	0.8693	0.8431	0.8592
Ours	**0.8806**	**0.8803**	**0.8810**	**0.8801**	**0.8806**

**Table 3:** Classification results of ablation studies. The bold values represent the best values.

Method	Accuracy	Precision	Sensitivity	Specificity	F1-Score
ResNet-50	0.8572	0.8474	0.8693	0.8431	0.8592
ResNet-50&CA	0.8617	0.8550	0.8594	0.8542	0.8602
ResNet-50&IB	0.8609	0.8597	**0.8814**	0.8553	0.8689
Ours (full)	**0.8806**	**0.8803**	0.8810	**0.8801**	**0.8806**

## Data Availability

Due to privacy and institutional restrictions, the datasets analyzed during the current study are not publicly available but are available from the respective authors upon reasonable request.
